# Prolonged Evaluation of COVID-19 Vaccine Effectiveness in Healthcare Workers at a Public Tertiary Hospital: A Retrospective Cohort Study

**DOI:** 10.1590/0037-8682-0219-2025

**Published:** 2026-03-30

**Authors:** Guilherme Silveira Castro, Yasmim Colins Silva de Oliveira, Michel Morais Marques Sawan, Marcela Padovan Prado Budoia, Janaína Soares, Mateus Rennó de Campos, Felipe Santos de Carvalho, Gilberto Gambero Gaspar, Benedito Antônio Lopes da Fonseca, Leandro Machado Colli, Antonio Pazin-Filho, Fernando Bellissimo-Rodrigues

**Affiliations:** 1 Universidade de São Paulo, Faculdade de Medicina de Ribeirão Preto, Ribeirão Preto, SP, Brasil.; 2 Universidade Federal de São Carlos, Departamento de Medicina, São Carlos, SP, Brasil.

**Keywords:** COVID-19, Vaccine, SARS-CoV-2, 2019-nCoV, Severe Acute Respiratory Syndrome Coronavirus, Vaccine effectiveness

## Abstract

**Background:**

Most studies evaluating COVID-19 vaccine efficacy and effectiveness were conducted over short follow-up periods due to the urgency of vaccine approval and rollout during the pandemic. This study aimed to assess the long-term effectiveness of COVID-19 vaccines among healthcare workers at a tertiary hospital.

**Methods:**

We conducted an open retrospective cohort study at Hospital das Clínicas de Ribeirão Preto, including healthcare workers employed between July 3, 2020, and February 8, 2022. A total of 7,385 workers were followed for 3,185,239 person-days. Three monovalent vaccines were administered during the study period: Coronavac™ (Sinovac), Covishield™ (AstraZeneca), and Comirnaty™ (Pfizer-BioNTech). Participants were categorized as unvaccinated, partially vaccinated, or fully vaccinated. The unadjusted relative risk (RR) of COVID-19 was calculated for vaccinated groups compared with the unvaccinated group.

**Results:**

For COVID-19 of any severity, the RR was 0.72 (95%CI: 0.59-0.89) among partially vaccinated and 1.30 (95%CI: 1.19-1.44) among fully vaccinated workers. For severe or critical cases, the RR was 0.75 (95%CI: 0.26-2.16) with partial vaccination and 0.14 (95%CI: 0.05-0.41) with full vaccination. For COVID-19-related deaths, the RR with full vaccination was 0.32 (95%CI: 0.03-3.09).

**Conclusions:**

The vaccines provided limited long-term protection against SARS-CoV-2 infection and mild COVID-19. However, protection against severe disease and death remained substantial despite the circulation of multiple variants.

## INTRODUCTION

COVID-19 is caused by the *Severe Acute Respiratory Syndrome Coronavirus* 2 (SARS-CoV-2)^1^, first reported in Wuhan, China, in December 2019^2^. The disease rapidly spread worldwide, resulting in more than 676 million confirmed cases and 6. 8 million deaths between January 2020 and March 2023^3^. In Brazil, over 37 million cases and more than 699,000 deaths were recorded during the same period^4^.

Since its emergence, SARS-CoV-2 has been the subject of extensive epidemiological and molecular research to clarify its transmission dynamics, genetic diversity, clinical impact, and the effectiveness of interventions, particularly vaccination. Viral genetics and evolution have remained central areas of investigation since the publication of the first viral genome^2^ ^,^ ^5^.

The rapid development and global distribution of COVID-19 vaccines marked a major milestone in modern medicine. The swift global spread of SARS-CoV-2 required an improved understanding of transmission patterns and preventive strategies to inform public health responses^6^. Multiple vaccines based on different technological platforms were developed. Following widespread vaccination, global declines in SARS-CoV-2 infections, severe cases, hospitalizations, and deaths were observed^7^ ^,^ ^8^. 

Vaccination has been a key public health measure in controlling the pandemic. Between January 22, 2020 and March 10, 2023, over 500 million vaccine doses were administered in Brazil^4^. During 2021-2022, vaccines authorized for use in Brazil included Coronavac^TM^ (inactivated whole-virus), AstraZeneca’s ChAdOx1-S (viral vector) (Covishield^TM^), and Janssen’s Ad26.COV2.S (viral vector), and Pfizer’s BNT162 (RNA) (Comirnaty^TM^)^9^ ^-^ ^13^. These vaccines demonstrated effectiveness in preventing infection and reducing COVID-19 severity, particularly severe acute respiratory syndrome^14^ ^,^ ^15^.

However, vaccine effectiveness is influenced by multiple factors, including host characteristics (e.g., age, prior infection, immune status, and comorbidities), demographic conditions (e.g., regional virus prevalence and vaccination coverage), vaccine-related factors (e.g., type of vaccine, number of doses, and dosing interval), and viral evolution (e.g., emergence of new variants with immune escape potential)^16^. 

Effectiveness also varies across population groups. Healthcare workers represent a high-risk group due to occupational exposure to SARS-CoV-2 and higher seroconversion rates following infection^17^ ^-^ ^20^. In addition to vaccination, appropriate use of personal protective equipment (PPE) and adherence to hand hygiene remain essential for risk reduction in healthcare settings^16^ ^,^ ^21^. 

This study evaluated the long-term effectiveness of COVID-19 vaccines in preventing infection, severe disease, critical illness, and death among healthcare workers at a tertiary public hospital in Ribeirão Preto, São Paulo, Brazil. The study also described circulating SARS-CoV-2 variants in the region. 

## METHODS

### Study design

This was a longitudinal, observational, and retrospective open cohort study conducted at the Hospital das Clínicas of the Ribeirão Preto Medical School, University of São Paulo (HCFMRP-USP), with healthcare workers employed during the COVID-19 pandemic from July 3, 2020, to February 8, 2022. The study assessed vaccine effectiveness with respect to the incidence density, severity, and mortality associated with COVID-19. This research is part of a larger project titled Uncovering New Coronavirus-Encoded Recourses, UnCoVER-Brazil.

### Study population

The study population included virtually all healthcare workers employed at the HCFMRP-USP during the study period, except those meeting the exclusion criteria. All healthcare workers were followed-up from July 3, 2020, or from their admission to the job if later than the study start date, until February 8, 2022, or until job discontinuation for any reason (censure). In the analysis, we did not distinguish between participants who were followed-up until the end of the study and those with censored follow-up.

We excluded professors, undergraduate and graduate students, and outsourced professionals from the study population due to limited continuity of service, high turnover, and lack of standardized data within the hospital’s information systems.

We analyzed a subpopulation of the study in greater depth, consisting of healthcare workers whose nasal samples were collected and stored at the study facility, thereby making them available for SARS-CoV-2 genotyping.

### Ethical considerations

This study was approved by the Institutional Review Board in March 2023. The requirement for informed consent was waived.

### Data collection

Data collection began in February 2023 via the ATHOS electronic health record system at HCFMRP-USP. Additional data regarding case notifications, vaccination history, and employment records were obtained from the Hospital Epidemiological Surveillance Center (NVEH) and the Information and Analysis Center (CIA). The data were recorded using the REDCap platform. Sampling was based on convenience.

Confidentiality was maintained through anonymized records. Only the date of employment, COVID-19 notification, vaccine administration, and RT-PCR results were accessed. Registration numbers were used to identify the participants, and all data were handled in accordance with the General Data Protection Law.

In the study design, the person-time concept was adopted to account for the time each individual remained at risk during the study period, as each group was assumed to have a different risk of SARS-CoV-2 infection. Accordingly, an individual could contribute to different groups (unvaccinated, partially vaccinated, and fully vaccinated) in units of person-days, depending on the timing of vaccine dose administration until the occurrence of a positive RT-PCR test result, hospital discharge, termination of employment, or the end of the study period.

**FIGURE 1:**
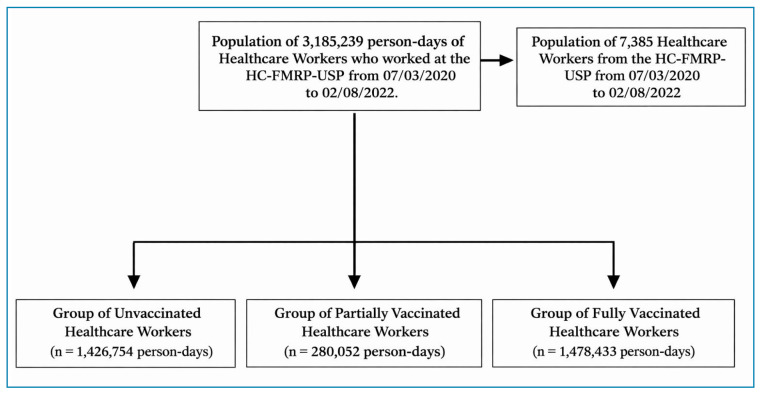
Sample of professionals participating in the study in person-day units.

A total of 7,385 healthcare professionals contributed 3,185,239 person-days between July 3, 2020, and February 8, 2022 (Figure 1). The cohort was categorized into three groups: unvaccinated (1,426,754 person-days), partially vaccinated (280,052 person-days), and fully vaccinated (1,478,433 person-days) groups.

The date of disease onset was defined as the date of symptom onset (if available) or the date of a positive RT-PCR test result. A 14-day window was used to account for the development of immunity, as studies have shown no difference in infection risk between unvaccinated groups and those vaccinated within 0-13 days of their first dose^22^. 

Group definitions were as follows:

- Unvaccinated: No vaccine or within 13 days of first dose.
- Partially Vaccinated: ≥ 14 days after first dose up to 13 days after second dose.
- Fully Vaccinated: ≥ 14 days after second dose.

Each individual was tracked until the earliest of the following: COVID-19 diagnosis, symptom onset, or end of the study. Outcomes were analyzed using incidence density per 1,000 person-days (for infections) and per 1,000,000 person-days (for severe/critical cases and deaths). The RR was calculated directly, and an unadjusted Poisson model with a normal approximation to the log rate was used to estimate 95% confidence intervals. Comparisons were performed between the partially- and the fully-vaccinated against the unvaccinated group. Vaccine effectiveness was assessed both overall and by vaccine type. 

The data collected included vaccination dates and types, COVID-19 infection dates, RT-PCR results, disease severity, and outcomes for the whole study population. Sociodemographic, occupational, and clinical characteristics along with SARS-CoV-2 genotypes were available and collected only for the study subpopulation.

COVID-19 severity was classified according to the World Health Organization (WHO) criteria as critical, severe, or mild/moderate cases^23^. The most severe clinical condition recorded at any point during the follow-up period was used to classify each case.

Regarding vaccine effectiveness, incidence density was analyzed for each vaccine type and number of doses with respect to SARS-CoV-2 infection, severe/critical illness, and death. Vaccine effectiveness was calculated based on these data.

The vaccines used during the pandemic in Ribeirão Preto included CoronaVac (Sinovac), ChAdOx1-S (AstraZeneca), Ad26.COV2.S (Janssen), and BNT162 (Pfizer)^11^ ^,^ ^13^ ^,^ ^15^ ^,^ ^24^. During the vaccination campaign for healthcare workers at the HCFMRP-USP complex, priority was given to staff in units providing direct care to COVID-19 patients, with those assigned in the ICU and COVID-19 wards being the first to receive vaccines^25^.

**FIGURE 2:**
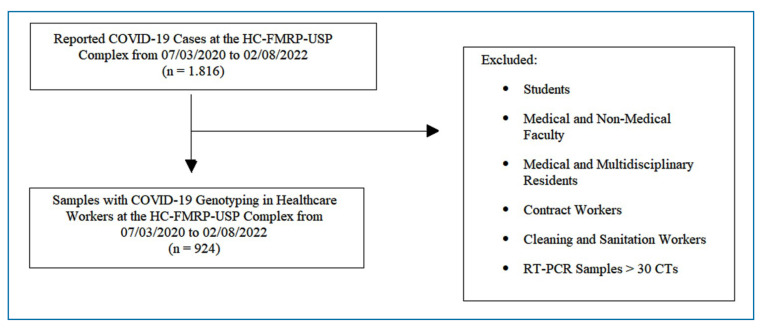
Sampling of Healthcare Workers with SARS-CoV-2 Genotyping

For SARS-CoV-2 genotyping in the study subpopulation, the project analyzed PCR-amplified respiratory secretion samples collected and stored at the study facility from healthcare workers who tested positive for the virus during the study period. A total of 924 SARS-CoV-2 samples from the healthcare workers were successfully genotyped (Figure 2). The genotyped subpopulation was analyzed comprehensively, including sociodemographic and occupational characteristics as well as prior comorbidities.

The initial samples were processed at the Virology Laboratory of the HCFMRP-USP. Sequencing was performed at the Sequencing Laboratory of the Ribeirão Preto Blood Center Foundation. RNA extracted from samples with RT-PCR cycle threshold (CT) values of < 30 was used for viral genome sequencing. The distribution of SARS-CoV-2 strains among healthcare workers was described based on epidemiological weeks throughout the study period.

To minimize bias in secondary data collection, a standardized data entry form was used on the REDCap Platform. Reports of SARS-CoV-2 infection from the NVEH were cross-referenced with the patients’ medical records to identify and resolve any discrepancies. In addition, meetings were held with the research team to standardize the medical record data collection procedures.

## RESULTS

A total of 3,185,239 person-days of health workers were analyzed, comprising 1,426,754 (44,8%) person-days in the unvaccinated group, 280,052 (8,8%) person-days in the partially vaccinated group, and 1,478,433 (46,4%) person-days in the fully vaccinated group. The mean follow-up duration was 193.2 days for unvaccinated persons, 37.9 days for partially vaccinated persons, and 200.2 days for fully vaccinated persons. In the unvaccinated group, there were 727 (40%) SARS-CoV-2 infections, 27 (1,5%) severe/critical cases of COVID-19, and three deaths (0,2%). In the partially vaccinated group, 103 (5,7%) infections were observed, with 4 (0,2%) severe/critical cases and no deaths. The fully vaccinated group recorded 986 (54,3%) infections, 4 (0,2%) severe/critical cases, and 1 (0,1%) death. The professionals were grouped according to the type of vaccine they received.

The study analyzed a population of 1,758,485 (46,4%) person-days of vaccinated healthcare workers divided into seven groups based on the vaccine and dose received.

The first group, vaccinated with the first dose of CoronaVac (86,855 person-days) (2,7%), recorded 32 (1,8%) SARS-CoV-2 infections, three (0,3%) severe/critical cases, and no deaths. In the second group, vaccinated with a second dose of CoronaVac (1,045,226 person-days) (32,8%), 686 (69,6%) infections, three (0,2%) severe/critical cases, and one (0,1%) death were recorded. The third group, who received AstraZeneca’s (Covishield^TM^) as first dose (189,299 person-days) (5,9%), recorded 68 (3,7%) infections, one (0,1%) severe/critical case, and no deaths. The fourth group, vaccinated with AstraZeneca’s (Covishield^TM^) second dose (406,592 person-days) (12,8%), reported 267 (14,7%) infections, one (0,1%) severe/critical case, and no deaths. The fifth group, vaccinated with the first dose of Jansen (24,795 person-days) (0,8%), had 27 (1,5%) infections, with no serious cases or deaths. The sixth group, vaccinated with Pfizer’s (Comirnaty^TM^) first dose (3,898 person-days) (0,1%), registered 3 (0,2%) infections, with no serious cases or deaths. The seventh group, vaccinated with Pfizer’s (Comirnaty^TM^) second dose (1,820 person-days) (0,1%), had 6 (0,3%) infections and no severe cases or deaths. The incidence density for SARS-CoV-2 infection, severe/critical COVID-19 cases, and COVID-related deaths were calculated for each group. The RR for each outcome was calculated relative to that of the unvaccinated group (Table 1). Notably, the data described are crude, and the estimates are unadjusted.

**TABLE 1:** Incidence density of SARS-CoV-2 infection, severe/critical cases of COVID-19, and death due to COVID-19 according to vaccination status among healthcare workers from a tertiary-care university hospital in Brazil.

	SARS-CoV-2 infection		COVID-19 (severe/critical cases)		Death due to COVID-19	
Vaccination status	Incidence density per 1,000 person/days (95%CI)	RR (95% CI)	Incidence density per 10^6^ person/days (95%CI)	RR (95% CI)	Mortality per 10^6^ person/days (95%CI)	RR (95% CI)
**Unvaccinated**	0.50 (0.47-0.54)	-	18.92 (1.21-27.59)	-	2.10 (0.68-6.52)	-
**Partially Vaccinated with any vaccine**	0.36 (0.30-0.44)	0.72 (0.59-0.89)	14.28 (0.56-38.05)	0.75 (0.26-2.16)	0.00	-
**Fully vaccinated with any vaccine**	0.66 (0.62-0.70)	1.30 (1.19-1.44)	2.70 (0.24-7.20)	0.14 (0.05-0.41)	0.68 (0.10-4.80)	0.32 (0.03-3.09)
**Fully vaccinated with Coronavac™**	0.66 (0.61-0.71)	1.29 (1.16-1.43)	2.87 (0.26-8.90)	0.15 (0.05-0.50)	0.96 (0.13-6.79)	0.46 (0.05-4.37)
**Fully vaccinated with Covishield™**	0.66 (0.58-0.74)	1.29 (1.12-1.48)	2.46 (0.22-17.46)	0.13 (0.02-0.96)	0	-
**Fully vaccinated with Comirnaty™**	6.47 (2.90-14.45)	3.30 (1.48-7.33)	0	-	0	-

Partial vaccination was associated with a reduced risk of infection (RR = 0.72). In contrast, full vaccination, although associated with an increased risk of infection (RR = 1.3), was protective against severe disease and death. Among those who were fully vaccinated, a reduction in the risk of developing severe forms (Unadjusted RR = 0.14) and death (Unadjusted RR = 0.32) was observed compared with those who were not vaccinated. Partial vaccination also offered protection (Unadjusted RR = 0.75); however, the result was not statistically significant (95%CI: 0.26-2.16). 

In the genotyped subpopulation, we analyzed 924 health workers who were genotyped for SARS-CoV-2, 75.65% of whom were female and 24.35% were male. The average age was 42.12 years. The most represented occupations were nursing technicians (27.16%), nursing assistants (15.26%), nurses (13.64%), and administrative officers (14.61%), with other occupations comprising the remainder of the sample (Table 2).

**TABLE 2:** Description of the socio-demographic characteristics of the subgroup of healthcare workers whose SARS-CoV-2 viruses could be genotyped.

Variables	N (924)	%
**Sex**		
Female	699	75.65
Male	225	24.35
**Age**		
Mean	42.12	-
Standard deviation	11.04	-
Min	18	-
Max	74	-
**Ethnicity**		
White	813	88.08
Brown	65	7.04
Black	36	3.90
Asian	9	0.98
**Profession**	**924**	**100.00**
Social Worker	4	0.43
Nursing assistant	141	15.26
Pharmacy assistant	9	0.97
Nutrition assistant	32	3.46
Biologist	5	0.54
Biologist	4	0.43
Biomedical Scientist	7	0.76
Dentist	3	0.32
Nurse	126	13.64
Pharmacist	12	1.3
Physiotherapist	30	3.25
Speech therapist	3	0.32
Attending Physician	107	11.58
Nutritionist	7	0.76
Administrative Officer	135	14.61
Psychologist	8	0.87
Nursing Technician	251	27.16
Laboratory Technician	15	1.62
X-ray technician	20	2.16
Occupational Therapist	5	0.54
**Comorbidities**		
Asthma	23	2.49
Diabetes mellitus	63	6.82
Chronic cardiovascular disease (including AH)	123	13.31
Chronic hematological disease	3	0.32
Chronic liver disease	4	0.43
Neurological or neuromuscular disease	4	0.43
Chronic lung disease	4	0.43
Chronic kidney disease	4	0.43
High-risk pregnancy*	7	1.00
HIV/AIDS	1	0.11
Immunodeficiency/immunodepression	3	0.32
Neoplasm	8	0.87
Obesity	81	8.77
Puerperium	2	0.29
**Covid severity**		
Mild/Moderate	896	96.97
Severe/Critical	28	3.03
Death by COVID-19	2	0.22
**SARS-CoV-2 genotype**		
Original strain	384	41.56
Apha	5	0.54
Gamma	237	25.65
Delta	46	4.98
Omicron	116	12.55
Zeta	99	1.84
Unknown	37	4.00

Description of demographic characteristics (sex, age, and ethnicity), professional categories, prevalence of comorbidities, COVID-19 severity, mortality rates, and distribution of SARS-CoV-2 genotypes among the subgroup of healthcare workers whose SARS-CoV-2 viruses were successfully genotyped. *% of total females n=699.

**FIGURE 3:**
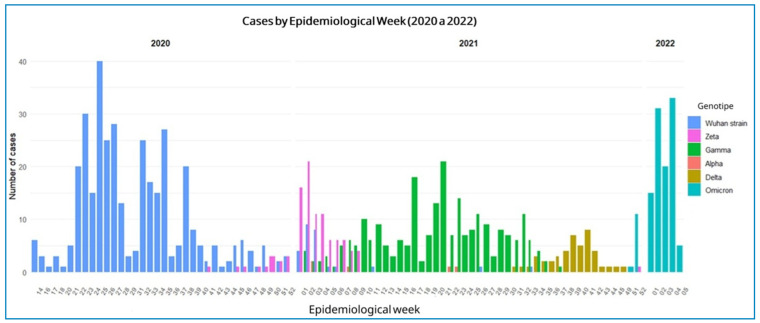
Distribution of SARS-CoV-2 sequenced genomes per epidemiological week isolated from healthcare workers from a tertiary-care hospital.

Regarding strain genotyping, the original SARS-CoV-2 strain was the most frequent, identified in 384 individuals (41.56%), followed by the Gamma (237 cases, 25.65%) and Omicron (116 cases, 12.55%) variants. The Delta variant was detected in 46 individuals (4.98%), Zeta variant in 99 (1.84%), and Alpha variant in five (0.54%). The genotype was unclear in 20 (2.16%) patients. Temporal evolution from 2020 to 2022 revealed that the original Wuhan strain was predominant in 2020, peaking between epidemiological weeks 20 and 30. The Zeta variant emerged at the end of 2020, followed by the dominance of the Gamma variant in the first half of 2021, the rise of Delta in the second half of 2021, and an increase in Omicron cases in early 2022 (Figure 3).

## DISCUSSION

The data show that COVID-19 infections occurred across all groups, regardless of vaccination status. Fully vaccinated workers had a higher RR of infection than unvaccinated workers, which may reflect several factors, including greater occupational exposure, increased confidence in vaccine protection leading to relaxation of individual protective measures, and longer cumulative exposure time. Healthcare workers, particularly those performing aerosol-generating procedures, such as intubation, are known to have a higher incidence of SARS-CoV-2 infection^26^ ^,^ ^27^. In addition, the emergence of new viral strains during the pandemic led to variable responses to SARS-CoV-2 infection and differences in severe and critical cases^28^. The longer duration of exposure among fully vaccinated workers may also have contributed to the higher observed infection rates. 

In contrast, the incidence density of severe/critical cases and COVID-19-related deaths was substantially lower among fully vaccinated professionals, demonstrating sustained protection against severe outcomes. This finding aligns with previous studies showing that vaccination significantly reduces disease severity, even when it does not fully prevent infection^29^.

Regarding the effectiveness of the different vaccines, the analysis highlights the role of vaccination in reducing severe disease and death. Notably, the second dose of CoronaVac (Sinovac) was associated with an 85% reduction in severe and critical cases (RR 0.15; 95%CI 0.05-0.50), and the second dose of AstraZeneca ChAdOx1-S with an 87% reduction (RR 0.13; 95%CI 0.02-0. 96). Although effectiveness varied across vaccines, all vaccines used during the study period contributed meaningfully to protection against severe disease. 

Regarding viral dynamics, the original SARS-CoV-2 strain predominated (41. 56%), followed by the Gamma (25. 65%) and Omicron (12. 55%) variants. In 2020, the Wuhan strain predominated, with peak cases between epidemiological weeks 20 and 30. The Zeta variant emerged at the end of 2020. In 2021, Gamma variant predominated during the first half of the year and was gradually replaced by Delta variant in the second half. In 2022, Omicron caused a sharp increase in cases, followed by a rapid decline.

These findings are consistent with Carvalho’s descriptive study of genotyping in Ribeirão Preto-SP^30^, which reported predominance of the original SARS-CoV-2 strains in early 2020 and a marked shift toward Omicron at the end of 2021 and beginning of 2022. This shift may partly explain the increased incidence of SARS-CoV-2 infection among fully vaccinated workers who received Pfizer BNT162 or AstraZeneca ChAdOx1^31^.

Several limitations should be considered. Selection bias may have occurred because the cohort included only healthcare workers treated within the HCFMRP-USP complex; cases diagnosed, hospitalized, or managed elsewhere may not have been captured. The high proportion of asymptomatic SARS-CoV-2 infections among healthcare workers may also have influenced vaccine effectiveness estimates^32^. Additionally, the inclusion and exclusion criteria limit generalizability to other healthcare settings. As with all secondary data analyses, underreporting and data inconsistencies are possible. Observations were not independent, as most participants contributed person-time to multiple vaccination categories. This was not accounted for in the aggregate statistical analysis.

Furthermore, the results are based on crude, unadjusted data and should be interpreted cautiously due to potential confounding. Temporal bias may also have influenced the findings, given the dynamic evolution of the virus, emergence of new variants, fluctuations in the local epidemic curve in Ribeirão Preto, and differences in vaccination coverage between healthcare workers and the general population. Successive pandemic waves, driven by variants with differing transmissibility and severity^30^, may have affected outcome patterns. Changes in adherence to PPE among vaccinated individuals may have further influenced exposure risk. Further investigation is warranted, particularly regarding the need for booster doses and the impact of circulating variants. Continued epidemiological surveillance and evaluation of vaccine effectiveness remain essential to inform public health strategies. 

Overall, the data reinforce previous evidence that the vaccines used during the study period provided limited long-term protection against SARS-CoV-2 infection and mild disease. However, two years after vaccination, they continued to confer protection against severe and critical COVID-19 and related mortality, despite shifts in circulating variants. 
